# Supplementary material for: Evaluating rural sports tourism competitiveness: A framework applied to Shandong province
Source: PLoS One. 2025 Jun 3;20(6):e0323868. doi: 10.1371/journal.pone.0323868 (PMC12132962; doi:10.1371/journal.pone.0323868)
Supplement: Attachment — Submitted filename: Response to Reviewers 4.docx [file pone.0323868.s003.docx]

**I am most grateful to you for your valuable suggestions regarding this paper. I have used the highlighting function to indicate the revisions made to the manuscript.**

1. The current title, "Rural Sports Tourism Competitiveness in Shandong Province," suggests a case study focused exclusively on Shandong Province, which may inadvertently limit the perceived generalisability of the study's findings. To better reflect the broader implications and applicability of the research, the title could be revised to emphasise the theoretical framework, methodology, or insights derived from the study. For instance, incorporating terms like "Framework," "Model," or "Evaluation" could highlight the study's contribution beyond a specific geographic context.

**Response: Thank you for your valuable comments. We understand your concern about the universality of the research results, but we chose to keep the current title "Rural Sports Tourism Competitiveness in Shandong Province" based on the following considerations: First, the title clearly reflects the core content and geographical background of the study, which makes it easier for readers to quickly understand the focus of the study; second, although Shandong Province is used as a case study, the niche theory framework and five-level comprehensive evaluation index system constructed in the study have wide applicability, and we further emphasized its theoretical contribution and methodological significance in the text. At the same time, we more clearly explained the universal value of the study in the introduction and conclusion, for example, by comparing the applicability of other regions, highlighting the promotion potential of the research results in a broader geographical context. (Please see lines 34-36, 802-834)**

2. The Introduction section requires significant improvements to enhance its clarity and comprehensiveness.

The second paragraph of the Introduction summarises previous research on tourism destination and sports tourism competitiveness. However, the discussion is overly concise and lacks sufficient detail to establish a solid foundation for the study. For example, the cited studies are briefly mentioned without adequately elaborating on their methodologies, key findings, or limitations. Expanding this section to provide a deeper discussion of the existing literature would better contextualise the research. Furthermore, incorporating more recent and region-specific studies on rural sports tourism competitiveness would demonstrate the relevance of the research and situate it within the broader academic discourse. This elaboration would strengthen the theoretical background and provide a clearer connection between the reviewed literature and the current study.

The research gap is unclear and requires explicit articulation. While the manuscript highlights that studies on rural sports tourism competitiveness are relatively limited, it does not sufficiently explain how the current study addresses these gaps or contributes to advancing the field. To strengthen this aspect, the authors should clearly identify the shortcomings in previous research, such as the lack of comprehensive evaluation frameworks or the limited focus on rural sports tourism, and explicitly state how the proposed five-tier evaluation index system and the use of niche theory provide a novel and meaningful contribution. This would clarify the rationale for conducting the study and emphasise its significance.

By addressing these issues, the Introduction would provide a more robust justification for the research and improve the reader's understanding of its objectives and potential contributions.

**Response: Thank you for your valuable comments. We have expanded the literature review, discussed in detail the research methods, core findings and limitations of tourism destination and sports tourism competitiveness, and supplemented the latest research on rural sports tourism competitiveness, especially the results of specific regions. At the same time, we have identified the shortcomings of existing research and emphasized the contributions of this study. (Please see lines 65-70, 85-107, 111-113)**

3. The Introduction would benefit from a clearer justification for focusing specifically on Shandong Province. While rural revitalization is highlighted as a national priority, the manuscript does not adequately explain why this province was chosen as the study context. Shandong’s rich cultural heritage, diverse natural landscapes, and historical significance as a hub for tourism and economic development make it a compelling case for examining rural sports tourism competitiveness. Explicitly linking these characteristics to the research objectives would enhance the contextual relevance. Furthermore, discussing how findings from Shandong might provide insights or serve as a model for other regions would strengthen the study’s broader applicability. Without this justification, the choice of Shandong risks appearing arbitrary, potentially weakening the manuscript’s overall contribution.

**Response: Thank you for pointing out the need for clarification. In the research area section, we explained the reasons for choosing Shandong Province, but at the same time, we will combine your opinions to further explain the advantages and representativeness of Shandong Province. In addition, in the theoretical significance and its extension section, by comparing other regions at home and abroad, we emphasized that the research results of Shandong Province can provide reference for other regions. (Please see lines 395-401, 405-406, 802-834)**

4. The Theoretical Basis section would benefit from a clearer distinction between the theoretical foundations and the methodological approaches to improve clarity and organisation. Currently, the discussion of niche theory as an ecological framework is well-developed, providing a solid foundation for understanding the theoretical underpinnings of the study. However, the subsequent introduction of formulas and their application to rural sports tourism begins to blend theoretical explanation with methodological details, potentially confusing readers about the purpose of this section.

To address this, the theoretical basis should focus exclusively on explaining the key concepts of niche theory—such as niche strength and niche overlap—in the context of rural sports tourism. This would include a conceptual explanation of how these constructs relate to the competitiveness of cities within the rural sports tourism ecosystem. Discussions about their ecological significance, relevance to tourism studies, and alignment with the research objectives should remain in this section.

The formulas and calculations, along with their application to the evaluation of rural sports tourism, should be moved to the Materials and Methods section. This separation would not only improve the structure of the manuscript but also allow for a more detailed and focused explanation of the methodological approach in its appropriate section. By making this distinction, the manuscript would present a clearer narrative, ensuring that readers can more easily follow the progression from theory to practical application.

**Response: Thank you for your suggestion. We understand your suggestion to clearly distinguish between theoretical explanations and formula descriptions, and have optimized the research. In the "3.1 Theoretical Basis" section, we explained in detail the core concepts of niche theory (such as niche intensity, niche overlap) and their application in rural sports tourism, and briefly explained the role and theoretical background of the formula to ensure that the theoretical part is both in-depth and clear. At the same time, the specific calculation steps and detailed application of the formula are expanded in the "3.2 Research Methods" section to avoid the theoretical part being too lengthy or confusing. (Please see lines 118-126, 140-142, 150-153, 368-392)**

5. The Methods section would benefit from a more detailed explanation of the AHP calculation steps, as the lack of citations or elaboration on the methodology leaves readers unable to fully understand or replicate the process. While the manuscript acknowledges that the Analytic Hierarchy Process (AHP) is context-dependent, this does not justify omitting either a brief explanation of the calculation steps or a citation to guide readers toward relevant resources.

To address this, the authors should either provide a concise overview of the key steps involved in AHP—such as constructing a pairwise comparison matrix, normalising the matrix, and calculating consistency—or cite authoritative sources that detail the procedure. This would ensure transparency and enable readers to assess the robustness of the subjective weighting method used. Additionally, clarifying how context-specific factors influenced the application of AHP in this study (e.g., the criteria used for pairwise comparisons and the selection of experts) would enhance the methodological clarity and reliability of the results. Without this information, readers may question the validity of the subjective weighting process and its integration with objective methods, potentially undermining the credibility of the evaluation framework.

**Response: Thank you for your valuable comments. We have optimized the AHP part of the document, supplemented the detailed description of the key steps, including building a hierarchy, judgment matrix, normalization and consistency test, and cited classic literature to ensure the transparency and replicability of the method. At the same time, we have detailed the specific operations of expert selection criteria and pairwise comparisons, and uploaded relevant data. (Please see lines 244-274)**

6. The Methods section provides a detailed description of the methodologies used, including the information entropy method, the least squares optimisation model, and the AHP approach. However, as a reviewer, I find it challenging to fully understand the methodological framework due to insufficient contextual explanation and the absence of citations to foundational literature. While the mathematical formulas are presented, the rationale for selecting these methods and their suitability for achieving the research objectives are not clearly articulated. This lack of clarity could make it difficult for readers to assess the robustness and relevance of the methodology.

To improve this section, the authors should provide a concise overview of why these specific methods were chosen and how they address the research questions. Additionally, they should include citations to standard references for these methodologies to guide readers who may not be familiar with them. For example, references explaining the application of the AHP approach or the entropy method in similar contexts would enhance the credibility and transparency of the study. Furthermore, a step-by-step explanation of how the methods were implemented in the specific case of rural sports tourism in Shandong Province would help readers better understand the process and evaluate the results. Without these improvements, the methodology section risks being inaccessible to readers who are not experts in these techniques.

**Response: Thank you for your valuable comments. We have optimized the methods section in the document, supplemented the rationale for the selection of methods, and explained in detail how these methods solve the research questions. In addition, we added step-by-step instructions for the implementation of the methods, such as the specific operation steps in the AHP method, the steps for data standardization in the information entropy method, and the process of weight integration in the least squares optimization model. (Please see lines 140-142, 150-153, 244-274)**

7. The Discussion section requires significant revision to adhere to standard academic conventions, enhance its scholarly impact, and provide clear implications.

First, the structure of the discussion does not follow the standard academic tradition. Typically, a discussion section should systematically address the key findings, compare them with existing literature, discuss their implications, and outline limitations and future research directions. However, this section appears more like a series of recommendations or strategies without sufficiently linking them to the study's results. A more structured approach is needed, beginning with an explicit interpretation of the findings in relation to the research objectives and followed by a critical comparison with previous studies.

Second, the discussion does not adequately compare the results of this study with findings from existing literature. For instance, while the study highlights the importance of resource endowment and infrastructure, there is no mention of how these findings align or contrast with prior research on rural tourism competitiveness. Including such comparisons would not only situate the study within the broader academic discourse but also strengthen its contribution by demonstrating how it confirms, extends, or challenges existing knowledge.

Third, the section lacks explicit implications. While some practical suggestions are scattered throughout, the implications for policymakers, practitioners, and academics are not clearly articulated. For instance, what specific policies should be enacted to support rural sports tourism development in Shandong? How can industry stakeholders utilise the study's findings to improve competitiveness? Furthermore, theoretical implications, such as how niche theory contributes to understanding tourism competitiveness, are entirely absent. These implications should be systematically discussed to enhance the relevance and impact of the study.

**Response: Thank you for your valuable comments. We have made significant revisions to the Discussion section to follow standard academic practices and enhance its academic impact. First, we restructured it, systematically explained the key findings, and compared them with existing literature, highlighting how this study confirms, extends, or challenges existing knowledge. Second, we clarified the practical significance of the research, provided specific suggestions for policymakers, practitioners, and scholars, and expanded the theoretical significance of the research through comparisons with domestic and international research. In addition, we discussed in depth the theoretical contributions of niche theory. Finally, we supplemented the limitations of the study and future research directions. (Please see lines 670-864)**

8. The Conclusion section lacks the standard academic structure needed to effectively summarise and contextualise the study. While it briefly discusses variations in competitiveness and proposes strategies for improvement, it fails to clearly link these findings to the study’s objectives or highlight its theoretical and practical contributions. The section does not adequately articulate the implications for policymakers or stakeholders, nor does it discuss how the study advances the application of niche theory in tourism research. Furthermore, it omits critical elements such as the study’s limitations and directions for future research, which are essential for transparency and academic rigour. To improve, the conclusion should concisely summarise the main findings, emphasise their implications, and include a discussion of limitations and potential areas for future investigation.

**Response: Thank you for your valuable comments. We have comprehensively revised the conclusion section to conform to the standard academic structure and enhance its theoretical and practical contributions. First, we succinctly summarize the main findings and clearly link them to the research objectives. Second, we systematically discuss the theoretical contributions of the research, while clarifying the practical significance of the research and providing specific suggestions for policymakers, practitioners, and scholars. In addition, we have added a discussion of the limitations of the research and future research directions to enhance the transparency and academic rigor of the research. (See lines 670-684)**

**I would like to express my gratitude once more for your invaluable contribution to the enhancement of this manuscript. Thank you!**
