# Supplementary material for: Evaluating rural sports tourism competitiveness: A framework applied to Shandong province
Source: PLoS One. 2025 Jun 3;20(6):e0323868. doi: 10.1371/journal.pone.0323868 (PMC12132962; doi:10.1371/journal.pone.0323868)
Supplement: Attachment — Submitted filename: Response_to_Reviewers_4_auresp_2.docx [file pone.0323868.s004.docx]

**I am most grateful to you for your valuable suggestions regarding this paper. I have used the highlighting function to indicate the revisions made to the manuscript.**

Comment 1

1.The current title, "Rural Sports Tourism Competitiveness in Shandong Province," emphasizes a specific region, which may limit its perceived relevance to readers outside this context. To better reflect the study’s broader methodological and theoretical contributions, I recommend revising the title to something like "Evaluating Rural Sports Tourism Competitiveness: A Framework Applied to Shandong Province." This adjustment highlights the framework’s potential applicability elsewhere while retaining the case study focus, aligning with your textual claims of wider significance and attracting a more diverse readership.

**Response: Thank you for your valuable comments. Your suggestions are very pertinent. After comprehensive consideration by our team, we decided to change the title to your suggestion: "Evaluating Rural Sports Tourism Competitiveness: A Framework Applied to Shandong Province.". (Please see lines 1-3)**

Comment 2

2. The "Determining subjective weights: AHP" subsection lacks detailed steps for the Analytic Hierarchy Process (AHP), stating only that they are context-dependent, which hinders transparency and replicability. I suggest including a concise summary of key AHP steps—such as hierarchy construction, pairwise comparisons, and consistency checks—along with a citation to a foundational source like Saaty (1980). If these details are already present elsewhere in the revised manuscript, ensure they are clearly integrated and accessible. Providing this clarity ensures readers can follow and replicate your subjective weighting process.

**Response: Thank you for your valuable comments. Thank you for your valuable comments. We have optimized the detailed steps of AHP (Analytic Hierarchy Process), supplemented the detailed description of key steps, including hierarchy construction, pairwise comparisons, and consistency checks, and cited Saaty's classic literature to ensure the transparency and replicability of the method. At the same time, we have uploaded the relevant detailed data of the judgment matrix and consistency check to the project "Figure", named "Comparison Matrix" and "Consistency Check" respectively. (Please see lines 245-288, 1088-1092)**

Comment 3

3. While the discussion provides practical recommendations, it lacks comparisons with prior studies and a clear explanation of niche theory’s theoretical contributions to rural sports tourism competitiveness. I recommend integrating comparisons with works like Wang & Zhu (2019) or Jia et al. (2022) to show how your findings align or differ, and explicitly detailing how niche theory advances tourism competitiveness research. These additions will situate your study within the broader academic discourse and underscore its theoretical and empirical significance.

**Response: We are very grateful for your suggestions.** **We highly appreciate your suggestion, and we have added comparisons with studies such as Wang & Zhu (2019) or Jia et al. (2022) to present our research findings and detail the theoretical contribution of niche theory to tourism competitiveness and rural sports tourism competitiveness (please see lines 849-868)**

Comment 4

4. The conclusion summarizes findings and strategies well but omits discussion of the study’s limitations and future research directions. I suggest adding a paragraph acknowledging limitations, such as the reliance on quantitative resource measures or potential subjective weighting biases, and proposing future steps, like testing the framework in other regions or incorporating qualitative data. Including these elements will enhance transparency, demonstrate academic integrity, and encourage further exploration of your work.

**Response: Thank you for your valuable comments. We highly appreciate your suggestions. Based on the points you raised, we have made targeted supplements to the discussion of research limitations and future research directions. (Please see lines 869-878)**

Comment 5

5. **The authors should avoid excessive dependence on AI tools, such as ChatGPT, when elaborating their manuscript. While AI can assist with drafting or structuring ideas, over-reliance may result in generic or shallow content that lacks the originality and critical depth required in academic writing. I encourage prioritizing human-led analysis and revisions to ensure the manuscript reflects your expertise and provides nuanced, authentic insights that meet scholarly standards.**

**Response: Thank you for your valuable comments. We completely agree with your point about avoiding over-reliance on AI tools and understand the importance of originality and critical depth in academic writing. During the writing process, we did use AI tools to assist in organizing ideas, but we always adhere to human-led analysis and revision, and all content has been deeply analyzed and revised multiple times by our team to ensure that it meets academic standards and reflects our expertise.**

**I would like to express my gratitude once more for your invaluable contribution to the enhancement of this manuscript. Thank you!**
