# Supplementary material for: Evaluating rural sports tourism competitiveness: A framework applied to Shandong province
Source: PLoS One. 2025 Jun 3;20(6):e0323868. doi: 10.1371/journal.pone.0323868 (PMC12132962; doi:10.1371/journal.pone.0323868)
Supplement: Attachment — Submitted filename: Response to Reviewer 4.docx [file pone.0323868.s005.docx]

**I am most grateful to you for your valuable suggestions regarding this paper. I have used the highlighting function to indicate the revisions made to the manuscript.**

**Comment 1**

1.The authors have indeed made notable progress by incorporating comparisons with prior studies like Wang & Zhu (2019) and Jia et al. (2022), as suggested. However, the discussion still predominantly lacks sufficient citations and detailed contrasts with relevant literature, which prevents readers from fully understanding how your findings confirm, differ from, or extend prior research.
To further enhance the academic rigour of your discussion section, I recommend clearly structuring it into three key components: (1) summarising your key findings succinctly, (2) explicitly comparing and contrasting your findings with those from existing studies, citing precise similarities or divergences, and (3) explaining the theoretical implications of these comparisons, particularly in relation to niche theory. Each of these components should be addressed separately and distinctly, accompanied by clear references to relevant literature. Increasing the depth of engagement with prior research will strengthen your manuscript’s theoretical contributions and help clarify the unique aspects your study brings to rural sports tourism competitiveness research.
For example, you could explicitly state how your conclusions about resource endowment align with or deviate from Wang & Zhu (2019), and whether your findings regarding niche overlap and municipal competitiveness offer new insights compared to Jia et al. (2022). Furthermore, explicitly identifying how your study fills existing theoretical gaps or addresses unanswered questions in niche theory literature would significantly strengthen your manuscript.
By following this approach, you will better highlight both the originality and theoretical contribution of your research.

**Response:** **Thank you for your valuable comments. We highly appreciate your suggestions. We have restructured 5 according to your suggestions on the discussion section and elaborated on the key parts you mentioned in 5.1 and 5.2.1. (Please see lines 683-703, 705-737)**

**Comment 2**

2. While the manuscript now appropriately discusses the results and limitations, it lacks a clearly structured subsection dedicated explicitly to Theoretical and Practical Implications. To enhance the paper's clarity and utility, I strongly recommend adding a separate subsection, clearly outlining:
Theoretical Implications: Specify how this research advances niche theory or contributes to theoretical understanding of rural sports tourism competitiveness. Clearly articulate the novel theoretical insights or extensions beyond existing studies, particularly highlighting how niche theory enriches the broader tourism competitiveness discourse.
Practical Implications: Provide concrete, actionable recommendations aimed at policymakers, tourism planners, and practitioners, clarifying exactly how the findings can inform policy decisions, marketing strategies, or infrastructure investments.
Presenting these implications explicitly in a dedicated subsection will significantly improve the manuscript’s value for both researchers and practitioners.

**Response:** **Thank you for your valuable comments. We highly appreciate your suggestions. Based on your suggestions, we have set the theoretical implications and practical significance you mentioned to 5.2.2, 5.2.3 and 5.3 respectively to clearly explain the questions you raised. (Please see lines 738-751, 752-768, 769-875)**

**Comment 3**

3. Although the authors briefly discussed limitations and future research directions within the "Discussion" section, the manuscript lacks a dedicated "Limitations and Future Research" subsection. This section is essential for explicitly acknowledging the boundaries of your research and guiding readers toward valuable next steps. To enhance clarity and rigor, I strongly recommend creating a separate subsection titled "Limitations and Future Research," clearly outlining limitations related to your data sources, methodological constraints, potential biases, and generalisability of your findings. Additionally, future research directions should be explicitly articulated, including suggestions on expanding geographic scope, integrating qualitative methods, or exploring other dimensions not fully addressed in your study. This dedicated subsection would significantly enhance the clarity and completeness of the manuscript, assisting readers in identifying research gaps and opportunities for further exploration.

**Response:** **Thank you for your valuable comments. We highly appreciate your suggestions. Based on your suggestions, we set "Limitations and Future Research" to 6 separately, and re-summarized and sorted out the limitations and future research respectively. (Please see lines 909-934)**

**I would like to express my gratitude once more for your invaluable contribution to the enhancement of this manuscript. Thank you!**
